# English Language Medical Schools in China: An Analysis of International Medical Graduates Practicing in the UK

**DOI:** 10.1177/23821205231163719

**Published:** 2023-03-14

**Authors:** Mohammed Ahmed Rashid, Victoria Smith, John Francis Mayberry

**Affiliations:** 1 4919 UCL Medical School, Faculty of Medical Sciences, University College London, UK; 2 Birmingham Medical School, College of Medical and Dental Sciences, 1724 University of Birmingham, Edgbaston, Birmingham, UK; 3 Department of Digestive Diseases, University Hospitals of Leicester NHS Trust, Leicester, UK

**Keywords:** Globalization, international, migration, language

## Abstract

**OBJECTIVES:**

From 2006, the Ministry of Education in China has approved universities to provide undergraduate medical training in English, targeting fee-paying international students. Students on these courses can face challenges in their clinical training, particularly in the domains of communication and professionalism. This study examines the proportion of doctors qualified from such medical schools who are currently listed on the UK medical register.

**METHODS:**

The UK General Medical Council register of medical practitioners was searched to identify doctors qualified from 33 Chinese medical schools who provide education in the English language.

**RESULTS:**

As of February 2022, 502 doctors whose primary medical qualification is from a university offering English language education in China were registered on the UK medical register. Four hundred twenty-five (84.7%) of these doctors were aged 39 and under, approximately double the proportion of doctors in this age bracket overall. Three hundred forty nine (69.5%) were staff grade and associate specialist doctors, 109 (21.7%) were doctors in training, 36 (7.2%) were on the General Practitioner (GP) register, and 20 (4.0%) were on the specialist register. Among doctors in training, the most common specialty areas were in general practice and psychiatry that are both facing recruitment shortages in the UK at present.

**CONCLUSION:**

A small but significant number of graduates whose medical training was in the English language in China are practicing medicine in the UK. These doctors are in younger age groups than the overall medical workforce, and are less likely to be in training, and specialist or GP posts. Among those in training, a high proportion are in GP and psychiatry training and could contribute to alleviating UK medical workforce shortages. Policymakers and educators should be mindful of the growing numbers of doctors qualified from these schools, and the additional support they may require considering the unique training environments they have encountered.

## Introduction

Medical education has become increasingly globalized in recent decades.^1^ As well as qualified doctors migrating for postgraduate training and continuing clinical practice, medical students have increasingly opted to move overseas for their basic medical education. This has included students from low- and middle-income countries moving to higher income countries as international students to study at some of the highest ranking universities in the world.^2^ However, it has also included citizens from all countries moving overseas to study at for-profit, private medical schools in various regions, including most prominently in Eastern Europe^3^ and the Caribbean.^4^

From 2006, the Ministry of Education in China has issued regulations for the “Provisions for Quality Control Standards on Undergraduate Medical Education in English for International Student in China”^5^ and during this period, many thousands of students from around the world have migrated to China to complete their basic medical education in English language schools.^6^ One of the attractions of training in China has been the low cost of the programs, including tuition fees and living costs.^7^

Previous studies examining the views of medical students on such programs has highlighted the significant challenges associated with studying medicine in English in China, particularly with regard to clinical placements, due to language, communication, and professionalism issues.^8–11^ A further challenge for these medical programs designed for international students in China is that they fall outside of the formal regulatory structures of the Working Committee for the Accreditation of Medical Education, creating further uncertainty about the oversight and scrutiny they receive in terms of their quality.^12^

A 2016 study based on a Freedom of Information request submitted to the UK General Medical Council (GMC) showed that a significant number of graduates from these medical schools are on the UK medical register.^13^ Of note, the study demonstrated that the majority of graduates from these schools were of South Asian descent, suggesting that this pathway is largely taken by those of non-Chinese backgrounds who move to China to access their basic medical education in English. There are no data addressing the clinical skills or capabilities of these graduates’ directly.

All doctors intending to practice medicine in the UK are required to be registered with the UK GMC. Doctors who hold registration but not a license cannot undertake any clinical work as UK law requires them to hold a license to practice. Doctors holding a primary medical qualification that is recognized by the GMC, who can demonstrate English language competence through scores on an accepted test, are then required to prove that they have appropriate clinical experience to practice in the UK. Although this can be done through direct entry to a specialist register or through a GMC-approved sponsor for further training, by far the commonest method is by passing the professional and linguistic assessments board test. This is an examination of language skills and medical competence in 2 parts. Part 1 can be taken overseas but part 2 must be taken at a dedicated clinical assessment center in the UK.^14^ While training across UK medical schools is fairly uniform and required to align closely with the GMC regulations,^15^ it has been noted that there is a significant degree of variation across medical schools in China.^3^

In light of significant medical workforce shortages in the UK, there is growing policy interest in routes for new medical graduates to enter the medical workforce from overseas. Uncertainty about the effects of the UK exiting the European Union on migration in the healthcare professions has particularly exacerbated this.^16^ There are also reasons why China is of particular interest in the UK context. Firstly, relations between the UK and China have intensified in recent years, with a commitment by the UK government to create a “Golden era” of co-operation with China in multiple sectors.^17^ Secondly, the region of the world most closely associated with parallel English language medical courses is Eastern Europe,^18^ with China being comparatively less recognized for this because of the relatively recent development of these programs. Finally, given the aforementioned findings about professionalism and communication shortcomings on these programs, and the growing importance of these non-technical skills in UK health policy,^19^ further training and support may be warranted in this group.

This study used the GMC Data Explorer tool from the UK GMC^20^ to investigate the number of graduates from English language medical schools in China who are currently registered medical practitioners in the UK. This tool was launched in 2020 and was therefore not available at the time this was previously examined in 2016.^3^ In identifying the trends of doctors entering the medical workforce from this pathway, including the specialty areas and career routes that they are pursuing, we hope to guide policymakers to make suitable arrangements and decisions with regard to regulation, training, and tailored support.

Our research question was as follows: How many doctors on the UK medical register qualified from English language medical schools in China, and what roles and specialty areas are they currently taking?

## Methods

We accessed the Chinese Ministry of Education website^21^ from London, UK, and followed a link on the official page to an English language website describing English language higher education opportunities in English within China (campuschina.org). We used the search function on this website to identify all programs using the keyword “Medicine” where the language of instruction was “English.” Of the 332 courses identified in this search, we identified all those describing 5- or 6-year courses in Clinical Medicine or MBBS. Courses in Traditional Chinese Medicine were not included. A total of 33 university courses were identified during this process.

The GMC Data Explorer was used to produce a search of the UK medical register in February 2022, using the filter function to identify doctors from the 33 universities. Searches were independently completed by 2 authors (MAR and VS), with no discrepancies noted between their findings.

Written informed consent was not viable in this study given that the GMC Data Explorer is entirely anonymized. The study was approved by UCL Research Ethics Committee (15443/005).

## Results

**Figure 1.**
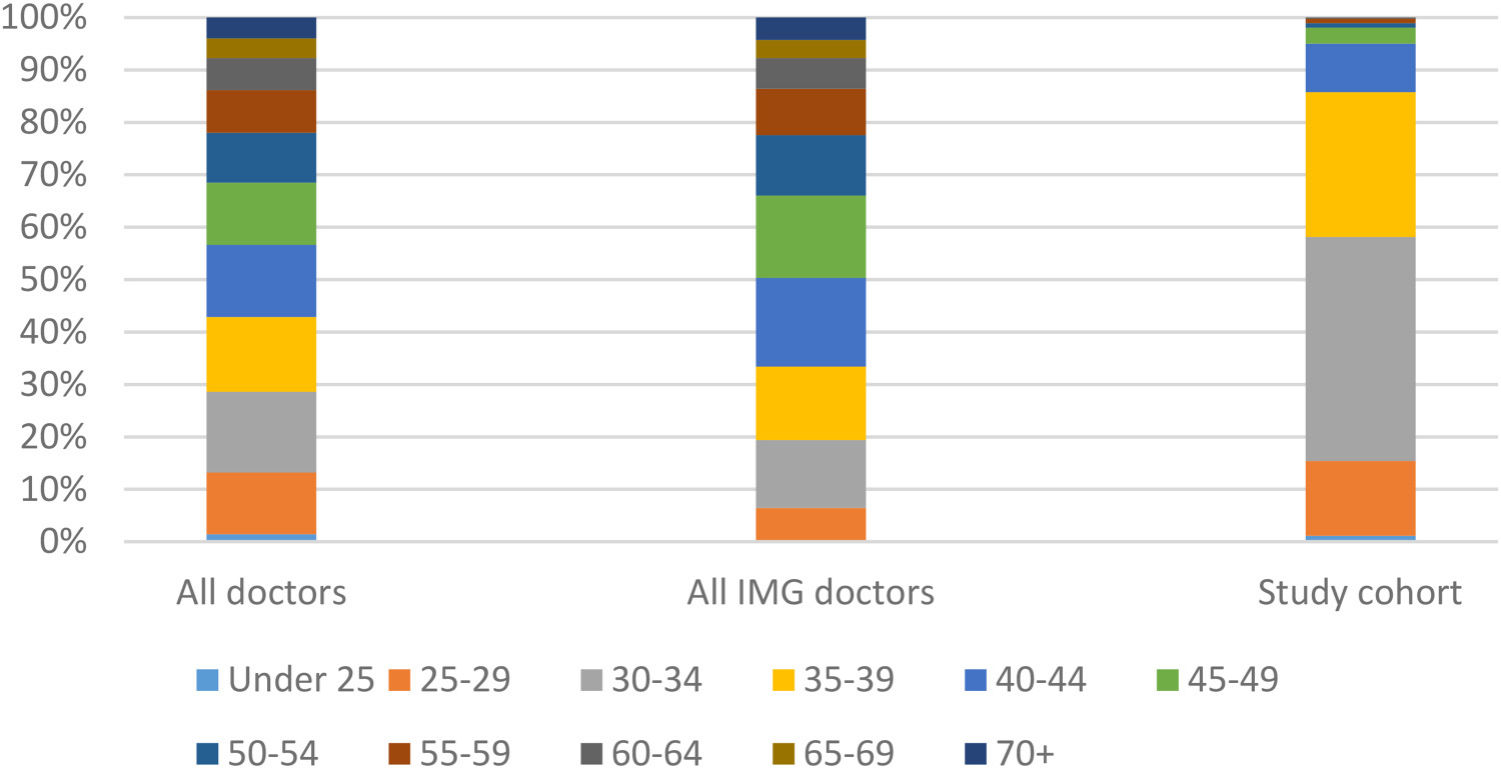
Doctors’ age bands on UK medical register.

A total of 502 doctors on the UK medical register had received their primary medical qualification from 33 universities in China offering English language training, of which 16 (3.2%) were registered without a current license to practice. Table 1 shows how this cohort of doctors compares to both the overall UK medical register and all international medical graduates (IMGs) on the register. The trend of male dominance in this group mirrors that of the overall register, and particularly IMGs. Strikingly, 425 (84.7%) doctors in this group were under 39 years of age, which indicates a much higher proportion of younger doctors than on the medical register and the wider IMG pool, consistent with the fact that these parallel English courses have only emerged in the last 2 decades. Figure 1 shows a more detailed spread of doctors’ ages in this cohort compared to the entire UK medical register and the overall population of IMGs, confirming that this group of doctors is considerably younger than the overall medical workforce in the UK.

**Table 1.** Summary of findings.

	**Full UK medical register**	**IMGs on UK medical register**	**Study cohort**
Registered with no license to practice	44 711 (12.7%)	18 276 (17.1%)	16 (3.2%)
Male	186 663 (53.2%)	65 740 (61.6%)	290 (57.8%)
Age under 40	150 507 (42.9%)	37 615 (35.3%)	425 (84.7%)
Specialist register	107 244 (30.6%)	26 456 (24.8%)	20 (4.0%)
GP register	79 729 (22.7%)	12 918 (12.1%)	36 (7.2%)
SAS doctors	105 668 (30.1%)	57 729 (54.1%)	349 (69.5%)
Doctors in training	67 254 (19.2%)	11 139 (10.4%)	109 (21.7%)
*Foundation training*	15 437 (23.0%)	374 (3.4%)	12 (11.0%)
*GP training*	13 979 (20.8%)	4756 (42.7%)	49 (45.0%)
*Psychiatry training*	3162 (4.7%)	825 (7.4%)	11 (10.1%)
Total	351 080	106 709	502

**Figure 2.**
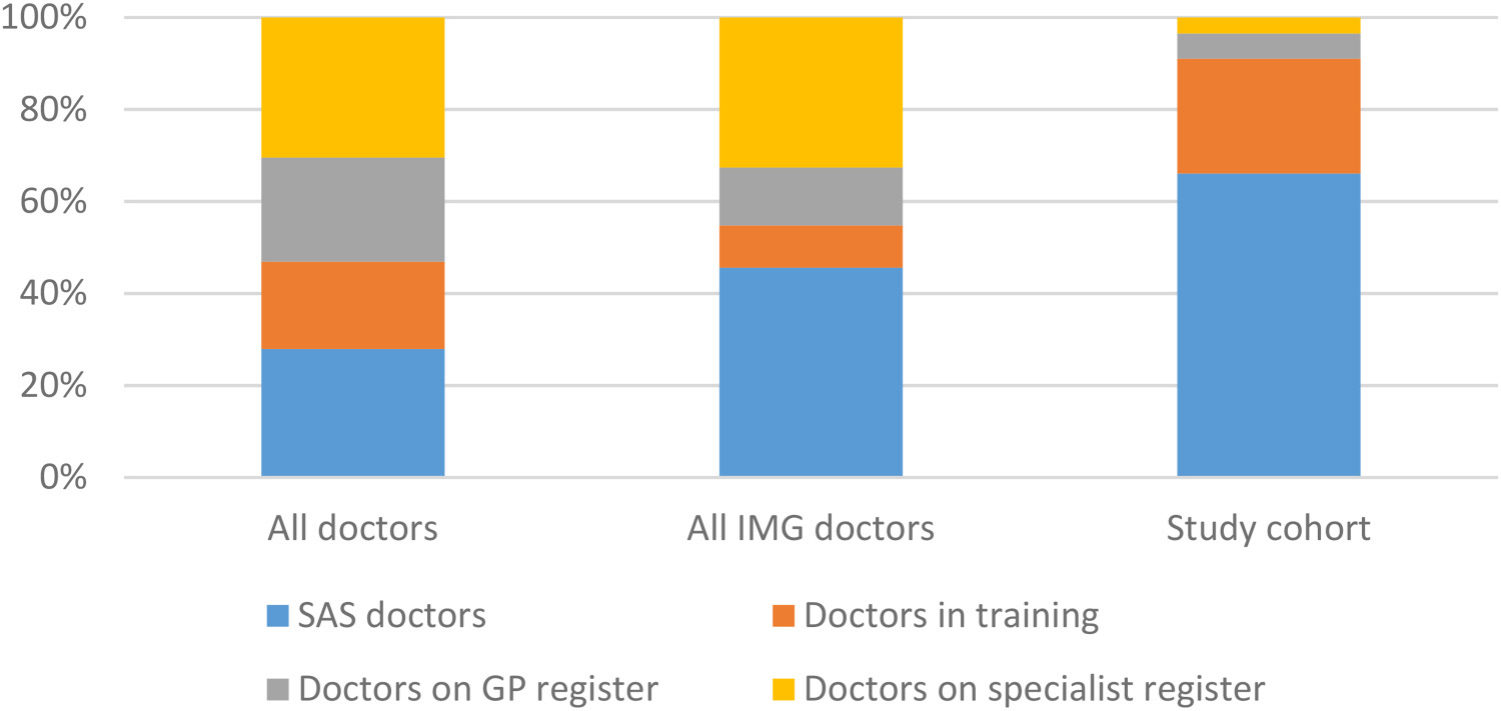
Doctors’ current roles on the UK medical register.

Figure 2 shows the distribution of doctors in this group according to current role, compared to the entire UK medical register. While the low proportion of doctors on the specialist and General Practitioner (GP) registers may be unsurprising due to the comparatively younger ages of this group, a significantly higher proportion of doctors are in staff grade and associate specialist (SAS) posts than those in formal training posts. SAS doctors are a diverse group with regards to their level of knowledge, clinical skills, training, performance, and needs. Given that they are outside of the mainstream medical training pathways, they are typically marginalized and given less priority than those if formal training pathways, and are often described as the “hidden heroes” of the UK National Health Service.^22^

Twenty (4.0%) doctors were on the specialist register, working across 7 different specialty areas, and 36 (7.2%) were on the GP register. Given that inclusion on these registers occurs at the end of postgraduate training, the comparatively low numbers are likely to be explained by the different age profile of this group of doctors.

Of the 109 doctors in training, 12 (11.0%) were in foundation training (the medical and surgical internship program for newly qualified doctors in the UK^23^), 49 (45.0%) were in GP training, 11 (10.1%) were in psychiatry training, and the remaining 37 were on other specialty training pathways. As shown in Table 1, the proportion of doctors in GP and psychiatry training is higher than the overall pool of IMGs and considerably higher than the overall register.

## Discussion

This study demonstrates that as of February 2022, 502 doctors on the UK medical register qualified from English language medical courses in China. Although this represents only 0.1% of doctors on the register, it is significantly higher than the figure of 73 doctors from a comparable study conducted in 2016. Doctors in this group tended to be younger than the overall medical workforce, and more likely to be working as SAS doctors, a group that has historically been marginalized and undervalued.^24^ They were less likely to be on the GP register than the overall medical workforce, but those in training were more likely to be on GP training programs.

IMGs practicing in the UK are less likely to pass postgraduate examinations in various specialty areas that allow them to complete their training,^25,26^ more likely to obtain a less satisfactory outcome through postgraduate training compared with UK graduates,^27^ and are more likely to be complained about and be sanctioned after a Fitness to Practice investigation,^28^ leading to suggestions that raising the standards of English language competency is required.^29^ There has also been a growing recognition of the challenges facing IMGs in the medical workforce, including racism and discrimination, as well as the significant contributions they make to the UK National Health Service.^30^

General practice in the UK is facing particularly acute workforce shortages,^31^ and low numbers of UK graduates enter GP training. Although the number of doctors in this study on the GP register is significantly lower than the overall UK medical workforce, the proportion of doctors in training pursuing GP was higher than the overall UK trainee doctor workforce and the overall pool of IMG doctors in training. This suggests this cohort of doctors could go on to make an important contribution to an under-resourced area of medicine in the UK, although IMGs in GP training are known to face particular challenges, including in Membership of the Royal College of General Practitioners assessments,^32^ which is required to attain a certificate of completion of training and practice as a GP independently in the UK.

As Figure 1 shows, the doctors in this study are significantly younger than the overall medical register, and the overall pool of IMGs in the UK. This is consistent with the fact that this new pathway was made possible only from 2006 onwards by the Chinese government.

The strength of this study lies in its systematic identification of English language medical courses from an official channel linked by the Chinese Ministry of Education, and the organized nature in which graduates from these institutions were located on the UK medical register. An important limitation of this study is that it is not possible to distinguish Chinese graduates from these universities compared to those from other countries, given that some of these universities offer parallel medical courses in English and local Chinese languages. Examination of ethnicity is not currently a function of the GMC Data Explorer tool. However, a previous study that was able to examine ethnicity confirmed that the majority of graduates from these schools are not ethnically Chinese. A further limitation is that there may be other Chinese universities offering English language medical training that were not identified from our search. Despite the webpage CampusChina.org using an official Government website link, there is a lack of information about its exact association with formal agencies. The impact of this is that our study is likely to underestimate the total number of graduates from these programs.

Although some research has examined aspects of the study environments that these students encounter, additional in-depth studies would help to provide further comparisons to other global contexts. Further research in this area could include studies examining graduates from these schools who practice in the UK in different ways to examine the backgrounds of individual doctors, including qualitative research to understand their experiences of transitioning to the UK after studying medicine in China, building on emerging work that has examined this.^33^ It could also examine differences between graduates from different individual schools, including for example, comparisons by tuition fee bands. Given the high number of doctors working as SAS doctors, further studies could also explore the career choices and pathways of this group in more detail, especially considering this group has been historically marginalized and often referred to as the “forgotten tribe.”^34^ Exploring further avenues to gain further information about Chinese medical schools and especially international student programs may also be considered. This study has additional implications for policymakers and regulators, who may consider whether current processes recognize that not all graduates from Chinese universities are ethnically Chinese and may have faced unusual and challenging circumstances in their training, as well as for educators, who may wish to tailor the training opportunities and pathways for doctors in this group. These may, for example, include prolonged induction programs and tailored support in the domains that have been noted to be challenging in this setting, such as communication and professionalism.

The COVID-19 pandemic has caused global disruption and this has been particularly noteworthy for international higher education and student mobility. International students studying in China have faced particularly challenging social and psychological circumstances^35^ and many have faced prolonged periods of remote learning, including from overseas. As such, the legacy of this period will be particularly important for educators and policymakers to track in the years ahead.

## Conclusion

A small but growing number of doctors qualified from English language medical courses in China are registered to practice medicine in the UK. Currently, these doctors are in younger age groups than the remainder of the medical workforce, and more likely to be SAS doctors than specialists, GPs, or trainee doctors. Given that doctors qualified in these medical schools are known to face particularly challenging circumstances from a linguistic and communication perspective, policymakers and educators may wish to monitor the increasing numbers of doctors entering the workforce on this pathway and provide tailored support to enable them to enter and complete formal postgraduate training successfully.

## References

[1] Clark PF Stewart JB Clark DA. The globalization of the labour market for health-care professionals. Int Labour Rev. 2006;145(1-2):37-64.

[2] Tavakol M Dennick R. Are Asian international medical students just rote learners? Adv Health Sci Educ. 2010 Aug 1;15(3):369-377. 10.1007/s10459-009-9203-1 19816780

[3] Hodges BD Maniate JM Martimianakis MA Alsuwaidan M Segouin C. Cracks and crevices: globalization discourse and medical education. Med Teach. 2009;31(10):910-917. 19877863 10.3109/01421590802534932

[4] Eckhert NL. Perspective: private schools of the Caribbean: outsourcing medical education. Acad Med. 2010;85(4):622-630. 20354377 10.1097/ACM.0b013e3181d2aee1

[5] Chan WK Wu X. Promoting governance model through international higher education: examining international student mobility in China between 2003 and 2016. High Educ Pol. 2020; 33(3):511-530.

[6] He JJ Chiang SY. Challenges to English-medium instruction (EMI) for international students in China: a learners’ perspective: English-medium education aims to accommodate international students into Chinese universities, but how well is it working? English Today. 2016;32(4):63-67.

[7] Li Y Wang Y Wan X. An empirical study on the motives of foreign students studying in China for China's MBBS programs: taking the West China School of Medicine, SCU as an example. In Proceedings of the 2019 3rd International Conference on Education and Multimedia Technology, 2019 Jul 22, pp. 245-251.

[8] Chen Q.. The development of an international medical education program with an emphasis on English for specific purposes. Minnesota State University, Mankato (2013) Cornerstone: a collection of scholarly and creative works for Minnesota University, Mankato. Theses, dissertations and other capstone projects. Accessed November 15, 2020. https://cornerstone.lib.mnsu.edu/cgi/viewcontent.cgi?referer=https://scholar.google.co.uk/&httpsredir=1&article=1270&context=etds

[9] Rashid MA Xu L Nicholson JG Gill D. “Doctor, teacher, translator:” International medical students’ experiences of clinical teaching on an English language undergraduate medical course in China. Educ Health. 2020;33(1):20-23. 10.4103/efh.EfH_212_19 32859876

[10] Yang P. Compromise and complicity in international student mobility: the ethnographic case of Indian medical students at a Chinese university. Discourse: Stud Cultural Politics Educ. 2018;39(5):694-708.

[11] Yang M O’Sullivan PS Irby DM Chen Z Lin C Lin C. Challenges and adaptations in implementing an English-medium medical program: a case study in China. BMC Med Educ. 2019 Dec 1;19(1):15. 30626387 10.1186/s12909-018-1452-3 PMC6325837

[12] WCAME. Accessed January 04, 2023. http://wcame.bjmu.edu.cn/en_index.php.

[13] Mayberry JF. Doctors qualified from Chinese universities with “English parallel” courses registered with the General Medical Council. Educación Médica. 2016;17(1):16-19.

[14] NHS Employers. Working and training in the NHS: a guide for for international medical graduates. 2021. Accessed April 20, 2022. https://www.nhsemployers.org/sites/default/files/media/Working-and-training-in-NHS-2021_0.pdf

[15] General Medical Council. Standards, guidance and curricula. Accessed January 04, 2022. https://www.gmc-uk.org/education/standards-guidance-and-curricula

[16] Fahy N Hervey T Greer S, et al. How will Brexit affect health and health services in the UK? Evaluating three possible scenarios. Lancet. 2017;390(10107):2110-2118. 28965715 10.1016/S0140-6736(17)31926-8

[17] Jie Y. China is a crucial partner for Britain to prosper outside the EU. LSE Brexit. 26 Jan 2018.

[18] Mayberry JF. The development of medical education in Eastern Europe during the 20th century and the emergence of ‘English parallel’courses. Scott Med J. 2013;58(1):46-52. 23596029 10.1177/0036933013476774

[19] Royal College of Physicians. Improving teams in healthcare: Team communication. 2017. Accessed April 21, 2022. https://www.rcplondon.ac.uk/projects/outputs/improving-teams-healthcare-resource-3-team-communication

[20] General Medical Council. GMC Data Explorer. Accessed February 06, 2022. https://data.gmc-uk.org/gmcdata/home/#/

[21] The People’s Republic of China. Ministry of Education. Accessed February 06, 2022. http://en.moe.gov.cn/

[22] Phazey G Agius S Hayden J. SAS doctors’ perceptions of their role in the NHS. Br Med J. 2012;344:e2819

[23] UKFPO. United Kingdom Foundation Programme. 2022. Accessed April 21, 2022. https://foundationprogramme.nhs.uk/

[24] Iacobucci G. Rude behaviour towards SAS doctors is unacceptable, says GMC. BMJ. 2020;368:m84. 31919044 10.1136/bmj.m84

[25] Rushd S Landau AB Khan JA Allgar V Lindow SW. An analysis of the performance of UK medical graduates in the MRCOG part 1 and part 2 written examinations. Postgrad Med J. 2012;88(1039):249-254. 22331918 10.1136/postgradmedj-2011-130479

[26] Tyrer SP Leung W-C Smalss J Katona C. The relationship between medical school of training, age, gender and success in the MRCPsych examinations. Psychiatric Bull. 2002;26(7):257-263.

[27] Tiffin PA Illing J Kasim AS McLachlan JC. Annual Review of Competence Progression (ARCP) performance of doctors who passed Professional and Linguistic Assessments Board (PLAB) tests compared with UK medical graduates: national data linkage study. Br Med J. 2014;348:g2622 10.1136/bmj.g2622 PMC3990835 24742539

[28] Humphrey C Hickman S Gulliford MC. Place of medical qualification and outcomes of UK General Medical Council “fitness to practice” process: cohort study. Br Med J. 2011;342:d1817. 21467101 10.1136/bmj.d1817 PMC3071377

[29] McManus IC Wakeford R. PLAB and UK graduates’ performance on MRCP (UK) and MRCGP examinations: data linkage study. Br Med J. 2014;348:g2621 10.1136/bmj.g2621 PMC3990836 24742473

[30] Esmail A Simpson J. International medical graduates and quality of care. Br Med J. 2017;356:j574. 10.1136/bmj.j574 28167488

[31] Irish B Purvis M. Not just another primary care workforce crisis. Br J Gen Pract. 2012;62(597):178-179. 22520894 10.3399/bjgp12X635985 PMC3310012

[32] Remedios L Deshpande A Harris M. Helping international medical graduates (IMGs) to success in the nMRCGP. Educ Prim Care. 2010;21(3):143-144. 20515542 10.1080/14739879.2010.11493899

[33] Jiang Q Horta H Yuen M. International medical students’ perspectives on factors affecting their academic success in China: a qualitative study. BMC Med Educ. 2022;22(1):1-6. 35897064 10.1186/s12909-022-03597-z PMC9325947

[34] Claxton N Griffin L. A forgotten tribe: a survey of the experience of working as a non-consultant career grade psychiatrist. Psychiatr Bull. 2006;30(10):369-372.

[35] Alam MD Lu J Ni L Hu S Xu Y. Psychological outcomes and associated factors among the international students living in China during the COVID-19 pandemic. Front Psychiatry. 2021;12:707342. 34483997 10.3389/fpsyt.2021.707342 PMC8414650

